# Underfed Children

**Published:** 1899-11-11

**Authors:** 


### The Hospital

A Journal of

The Medical Sciences and Hospital Administration.

Vol. XXVII.—No. 685. EDITED BY SIR HENRY BURDETT, K.C.B., AND SOLOMON C. SMITH, M.D., M.R.C.P. [November 11, 1899.

### Underfed Children.

The London School Board has postponed for a fortnight the consideration of the report of its Sub-Committee on Underfed Children, and has thus given time, not only for the members of the Board itself to become fully acquainted with the facts of the case, but also for public opinion to be brought to bear upon the whole of the question at issue. This, as it is presented to the School Board, is merely whether the necessities of underfed children should be supplied at the expense of the ratepayers, as is recommended by the majority of the sub-committee, or by a better organisation of voluntary charity, as is recommended by the minority, and enforced in a letter from one of them, Sir Charles Elliott, the chairman of the Finance Committee of the Board, in a letter which appeared in the *Times* of November 2nd. In reality, it is far more complicated, and involves medical and physiological considerations which cover a very extensive area. The total number of "underfed children," in ordinary times, is estimated by the sub-committee at 55,050, or about 12 per cent of the whole number in attendance at the schools; and Sir Charles Elliott, speaking from intimate knowledge of his own district, the Tower Hamlets, says that the requirements of the case are therein fully met by private organisations in 66 schools, with a total of 6,672 children; doubtfully in 13, with 863 children; and insufficiently, or not at all, in 13 more, with 970 children. This, on the percentage above stated, appears to mean that about 1,450 necessitous children are at present fed in the district, and that the feeding agency need only be extended to 220 more in order to render its operations complete. It seems manifest that an enlargement by one-seventh of a work which is at present accomplished ought not to be a task beyond the powers of private organisations actually in existence. In the meantime, it is necessary carefully to consider the causes of the underfeeding, as to which the report is only hesitating and speculative. In what proportion is it due to poverty, and in what proportion to parental self-indulgence, indifference, and neglect? In what proportion of cases is the father of the underfed child out of work, or working for a wage insufficient to support his family, and in what proportion does he spend his earnings at the public-house? Under the former conditions charity may be well bestowed, assuming, of course, that it does not, as with the doles under the old Poor Law, come to be regarded as a legitimate supplement to insufficient earnings, and as a reasonable excuse for accepting them; but under the latter it would be an unmixed evil, certain to produce an ever growing demand, and certain also, in supplying it, fatally to undermine any remaining or rudimentary notions of parental duty and of honest self-respect. Charity to the children of delinquent parents should be steadily associated with sharp punishment of the parents themselves; and the idle or drunken father should pay, by a short term of hard labour and restricted diet, for the offence of leaving the breakfasts and dinners of his children to be provided either by charity or by the rates.

But a far more important question, and one which is primarily physiological rather than economical, is, "What are we really doing to 55,050 underfed children in trying to teach them?" The attempt is being made, and, as we are all reminded twice a year, it is being made at great expense. Is it not only too probable that it increases physical degeneracy, without exerting any real influence in the promotion of mental growth. If a child be underfed, is it not a matter of plain necessity that its expenditure in living should be restricted within the narrowest possible limits? To call upon it to learn as well as to grow is to increase this expenditure; and every family medical attendant is constantly advising the parents of delicate children, who are practically "underfed" by reason of defective assimilation, to defer their education until they are in sufficiently good physical condition to bear the additional strain which it produces. It is worth while to consider, in relation to the 55,050 children declared to be underfed, whether the operation of the Education Act may not be doing them considerable and even irreparable mischief, and doing it at great cost to the country. The physiologist knows quite well that the introduction of compressed facts does not mechanically expand the intellect; and the average Londoner may be excused for entertaining some doubt as to whether, judging from the manners and customs of the lower classes of the juvenile population, the operations of school teachers have been of any considerable benefit to the community. A great Archbishop is reputed to have said that, if he were compelled to choose, he would rather see Englishmen free than sober. For those who have to earn their living by manual labour, we would in like manner rather see good physical development, even at the cost of ignorance, than an ability to read gutter journalism, obtained by forcing an underfed brain at the cost of muscle and sinew.

